# A database of zooplankton biomass in Australian marine waters

**DOI:** 10.1038/s41597-020-00625-9

**Published:** 2020-09-08

**Authors:** Felicity R. McEnnulty, Claire H. Davies, Asia O Armstrong, Natalia Atkins, Frank Coman, Lesley Clementson, Steven Edgar, Ruth S. Eriksen, Jason D. Everett, J. Anthony Koslow, Christian Lønborg, A. David McKinnon, Margaret Miller, Todd D. O’Brien, Sarah A. Pausina, Julian Uribe-Palomino, Wayne Rochester, Peter C. Rothlisberg, Anita Slotwinski, Joanna Strzelecki, Iain M. Suthers, Kerrie M. Swadling, Mark L. Tonks, Paul D. van Ruth, Jock W. Young, Anthony J. Richardson

**Affiliations:** 1 CSIRO Oceans and Atmosphere, GPO Box 1538, Hobart, TAS 7001 Australia; 2 CSIRO Oceans and Atmosphere, Queensland Biosciences Precinct, St Lucia, QLD 4067 Australia; 3 CSIRO Oceans and Atmosphere, Indian Ocean Marine Research Centre (UWA), M097 35 Stirling Highway, Crawley, WA 6009 Australia; 4 School of Biological Sciences, The University of Queensland, St Lucia, QLD 4072 Australia; 5 Institute for Marine and Antarctic Studies, University of Tasmania, Private Bag 129, Hobart, TAS 7001 Australia; 6 Australian Institute of Marine Science, Townsville, QLD 4810 Australia; 7 Centre for Applications in Natural Resource Mathematics, School of Mathematics and Physics, The University of Queensland, St Lucia, QLD 4072 Australia; 8 School of Biological, Earth and Environmental Science, University of NSW, Sydney, NSW 2052 Australia; 9 Sydney Institute of Marine Science, 19 Chowder Bay Road, Mosman, NSW 2088 Australia; 10 South Australian Research and Development Institute – Aquatic Sciences, West Beach, SA 5024 Australia; 11 NOAA Fisheries - COPEPOD, Silver Spring, Maryland USA; 12 Australian Ocean Data Network, Integrated Marine Observing System University of Tasmania, Private Bag 110, Hobart, TAS 7001 Australia; 13 Project Manta, School of Biomedical Sciences, The University of Queensland, St Lucia, QLD 4072 Australia; 14 Scripps Institution of Oceanography, University of California, San Diego, La Jolla, California, 92093 USA; 15 Section for Applied Marine Ecology and Modelling, Department of Bioscience, Aahus University, 4000 Roskilde, Denmark

**Keywords:** Population dynamics, Zoology, Databases

## Abstract

Zooplankton biomass data have been collected in Australian waters since the 1930s, yet most datasets have been unavailable to the research community. We have searched archives, scanned the primary and grey literature, and contacted researchers, to collate 49187 records of marine zooplankton biomass from waters around Australia (0–60°S, 110–160°E). Many of these datasets are relatively small, but when combined, they provide >85 years of zooplankton biomass data for Australian waters from 1932 to the present. Data have been standardised and all available metadata included. We have lodged this dataset with the Australian Ocean Data Network, allowing full public access. The Australian Zooplankton Biomass Database will be valuable for global change studies, research assessing trophic linkages, and for initialising and assessing biogeochemical and ecosystem models of lower trophic levels.

## Abstract (summary)

**Table:** 

Measurement(s)	zooplankton biomass • organic material • planktonic material
Technology Type(s)	digital curation
Factor Type(s)	year of data collection
Sample Characteristic - Organism	zooplankton
Sample Characteristic - Environment	marine water body
Sample Characteristic - Location	Australia

Machine-accessible metadata file describing the reported data: 10.6084/m9.figshare.12651425

## Background & Summary

Zooplankton are the animal component of the plankton and are the primary link between phytoplankton and higher trophic levels. The term “plankton” is derived from the Greek word *planktos* meaning “to drift” and includes organisms capable of movement in water but unable to progress against currents. Zooplankton communities are highly diverse and almost all phyla are represented. They range from microzooplankton such as heterotrophic flagellates, foraminiferans and radiolarians, to metazoans such as crustaceans, chaetognaths, molluscs, cnidarians and chordates including salps and larval fish. Zooplankton are extremely abundant and one group, the copepods, may even outnumber insects in abundance^1^. The carrying capacity of marine systems – the biomass of exploited fish, squid and shellfish; the numbers of marine mammals, seabirds and sea turtles; and the diverse bottom-dwelling communities of fish and invertebrates– is influenced by the biomass of zooplankton. The Australian Zooplankton Biomass Database is focused on the meso- and macrozooplankton (0.2–20 mm and 2–20 cm, in size) traditionally sampled by devices that filter the plankton from the water directly at sea: towed nets (e.g. bongo nets and ring nets) and towed samplers (e.g. Continuous Plankton Recorder (CPR)). The majority of samples were collected by nets with mesh sizes 200–333 µm although meshes vary from 73–550 µm. More recently, optical devices such as the Optical Plankton Counter (OPC) and the Laser Optical Plankton Counter (LOPC) have been used to estimate zooplankton biomass and available data are also included.

Biomass is the most common metric used to measure the entire zooplankton community and can be measured in many ways and each with different units^2–4^. Zooplankton biomass can be calculated from the zooplankton weight (e.g. wet mass, dry mass or carbon mass), or zooplankton biovolume (e.g. displacement volume, settled volume). The biovolume- the volume of zooplankton (e.g. in mL) is converted to mass by assuming zooplankton are neutrally buoyant^5^ with a density of 1 g/cm^3^. When comparing biomass values, it is customary to standardise them to the volume of water filtered during the collection of the sample. To enable comparisons, this dataset also includes a conversion of the biomass values into carbon values using the standard units of µg Carbon per litre (µg C/L) which are equivalent to mg C/m^3^, with the exception of the optical generated data.

**Fig. 1:**
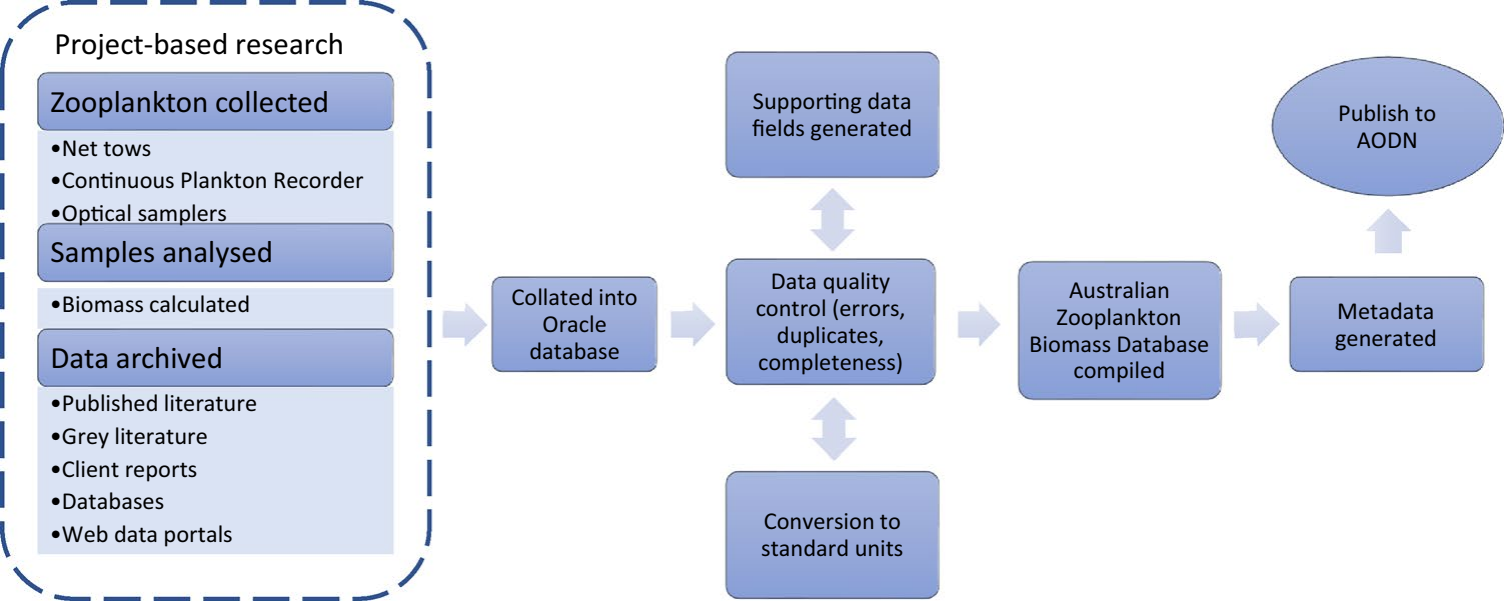
Flow diagram showing process of data entry into biomass database.

The Australian Zooplankton Biomass Database^6^ follows on from a series of datasets collated for the Australian marine region for zooplankton composition and abundance^7^, phytoplankton composition and abundance^8^ and chlorophyll a^9^, made freely available through the Australian Ocean Data Network (AODN) portal (https://portal.aodn.org.au/). Figure 1 is a flow diagram of the data acquisition and quality assurance and quality control processes used in collating the database.

**Fig. 2:**
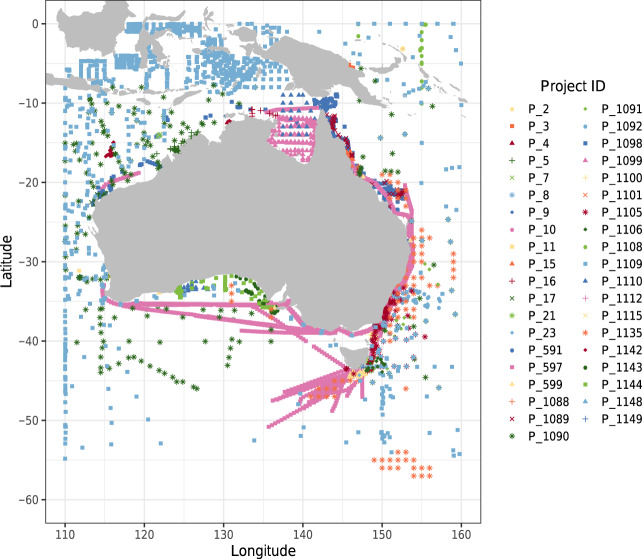
Sampling locations mapped by Project, see Online-only Table 1 for project details using project id.

Records collated here for zooplankton biomass largely cover the greater Australian region, including the Southern Ocean (Fig. 2). We compiled 39 datasets, containing 49187 records, from 1932 to the present. Of these, 11573 records are from net samples, 2256 from Continuous Plankton Recorder (CPR) samples and 35358 are from Optical Plankton Counter (OPC) and Laser OPC (LOPC) calculated biomass records. A metadata summary for the individual project data included in the dataset is shown in Online-only Table 1.

The biomass dataset can be used to: develop maps of zooplankton biomass; determine changes in zooplankton biomass over time or with oceanographic conditions; and to initialise and assess biogeochemical and ecosystem models^10^. The Australian Zooplankton Biomass Database is available through the AODN. The dataset is maintained by the CSIRO Data Centre and is updated with new records periodically and uploaded to the AODN. Researchers wishing to submit new data should contact the corresponding author or the AODN. A snapshot of the Australian Zooplankton Biomass Database as it is at the time of this publication has been assigned a DOI and will be maintained in perpetuity by the AODN^6^.

## Methods

Data held in the Australian Zooplankton Biomass Database have been collated from literature, active and retired researchers, consultancies, archives and databases. Only project data with the relevant corresponding metadata regarding collection location, date and methods have been included. Samples have been collected on research and commercial vessels in coastal and oceanic waters across a range of depths from the tropics to the sub-Antarctic, with estuarine waters excluded. Samples for zooplankton biomass measurement were collected in one of three ways in the current dataset. First, using towed nets^5,11,12^, with a variety of mesh sizes and diameters. Second, using the CPR^13^, which has an aperture of 1.61 cm^2^. Last, by using optical counting instruments (e.g. the OPC or LOPC)^14,15^, which were towed *in situ* and measure size and number of zooplankton via attenuation of a light or a laser beam.

**Fig. 3:**
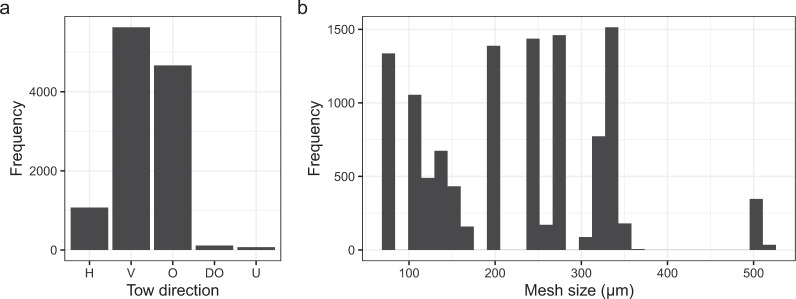
Summary of (**a**): Net-collected data records showing the frequency of the tow direction and (**b**): Range of net mesh sizes used. H = horizontal tow, V = vertical tow, O = oblique tow, DO = double oblique tow and U = tow direction unknown.

The collection strategy (sampling method, tow direction and time of day) used by individual projects has an important influence on the biomass of zooplankton collected. Many zooplankton vertically migrate diurnally, and larger ones can swim sufficiently to evade collection. This dataset includes samples collected by nets using different tow directions (vertical, horizontal and oblique, Fig. 3a). Vertical net sampling can mitigate effects of vertical migration if the entire water column is sampled such as in shallow coastal and shelf waters, but in oceanic locations vertical net drops are often stopped prior to the bottom, typically at about 200 m, missing deeper zooplankton. Oblique net tows sample diagonally through the water column between specified depths, and are often used to target larger, faster-swimming zooplankton. Horizontal net tows can be taken at the surface or at specific depths and are often used to target a specific feature of the water column (e.g. the deep chlorophyll maximum, the bottom of the mixed layer or an acoustically detected swarm) or to understand vertical migration. Samples in the dataset have been collected throughout the day and night and collection times are included where available. We have generated the variable: day_night, in the database to determine whether the sample was collected in the day or night to aid analysis of diurnal migration. This was estimated using the *crepuscule* function in the *maptools* package in R^16^, which compares the local time of the sample with the times of dawn and dusk for the date and latitude. Where bathymetric data were directly measured by the project it is provided by variable: bottom_depth_measured. To provide bathymetrical data for each record in the database, we determined the bottom depth based on latitude and longitude. Bathymetric depth estimates were obtained from the ETOPO1 bathymetry and topography database developed using an one Arc-Minute Global Relief Model^17^ using the *Marmap* package in R^18^, as the variable: bottom_depth_ETOPO_estimate.

The size of zooplankton targeted in net tows, whether micro-, meso-, or macro-zooplankton, is dependent upon the mesh size used, the diameter of the net, and the direction the net is towed, and the tow speed^19^. Finer-mesh nets generally collect more zooplankton and thus measure higher biomass because smaller species and more life stages are captured. However, finer-mesh nets (e.g. 100 µm) are more prone to clogging by phytoplankton and consequently can only be towed for short distances and at slow speeds, therefore sampling a smaller water volume. These smaller water volumes are appropriate for sampling microzooplankton and mesozooplankton such as copepods, but not ideal for sampling the rarer and larger macrozooplankton. This can be compensated for by increasing the filtering area of the net^20,21^. Coarser-mesh nets (e.g. 500 µm), often with larger diameters can be towed at higher speeds to capture larger, more-motile zooplankton such as euphausiids and amphipods, but miss the small zooplankton^22–25^. Coarse mesh nets are often towed obliquely and deployed in deep waters to target the larger zooplankton that can be strong vertical migrators which swim to the surface at night and sink deeper during the day. There is no one ideal net or mesh size to capture all zooplankton sizes.

Many net mesh sizes exist in the project datasets ranging from 73–550 µm mesh, Fig. 3b. Net types and dimensions also vary considerably among projects. Some nets can open and close to target particular depths, and some vertical nets (e.g. ring nets) are deployed free-fall (sampling going down) or hauled upwards (sampling going up). The various sampling gears and measurement methods used require that care should be taken when comparing biomass values across projects, while gear and methods are usually consistent within each project. In particular, mesh size is a major determinant of the magnitude of the biomass measurement. For example, McKinnon^26^ found the biomass of the 73 µm mesh fraction of the zooplankton was 2.3 times greater than the 350 µm mesh fraction collected in waters of the Great Barrier Reef and 3.6 times greater in waters off the Kimberley coast. The database includes the total zooplankton biomass as recorded by the individual projects, this may include sporadically high abundances of gelatinous species. For further information on how each project dealt with this component of the plankton, please consult the relevant project citation.

**Fig. 4:**
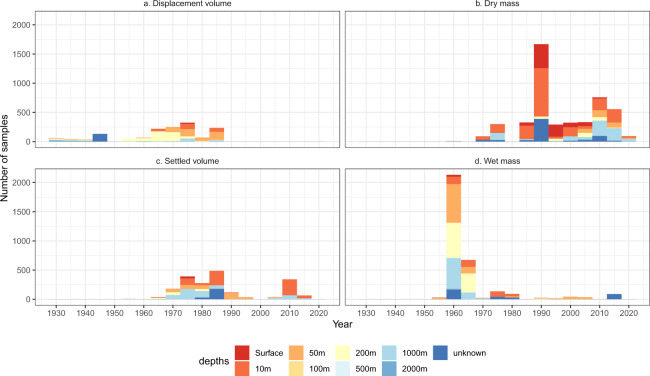
Histograms of the net-collected data records showing number of samples over the collection period for each biomass measurement method by sampling depth.

Biomass calculations require the volume of water filtered during the zooplankton collection; the volume is the product of the distance travelled and net mouth area. This can be obtained by a combination of the following variables: tow duration, calibrated flowmeter values, tow speed and/or sampling depth. Hence, a variety of calculations exist to determine the volume of water sampled^2,27–30^. The collection method and biomass measurement are included for inter-project comparisons (Online-only Table 1). For the Australian Zooplankton Biomass Database, the initial zooplankton biomass values were all converted into standard units of µg Carbon per litre (µg C/L) using accepted calculations^2,28,31–35^ for the zooplankton dry mass, wet mass, displacement volume or settled volume. The frequency of use of the different biomass measurement methods varies over time, Fig. 4.

Probably the most important effect of gear on the magnitude of zooplankton biomass is mesh size^25,36,37^. Comparisons of zooplankton biomass collected by different nets and samplers were summarised by Skjoldal^38^. Certainly the traditional standard 200 μm mesh net was used more in Australia in the past, but is now considered inappropriate for capturing the smaller zooplankton typical of oligotrophic tropical waters in much of the region^26,39^. In the analysis of the global COPEPOD dataset^40–44^, they found 333 μm mesh to be the most commonly used mesh size, so a factor was derived to convert all the other biomass records to 333 µm-equivalent values to enable comparison among datasets. It was noted that each mesh size did not offer a complete geographic coverage and that the use of 333 μm mesh was absent in their Southern Ocean data^33,40–44^. The mesh size in the Australian Zooplankton Biomass Database is highly variable and varies spatially as well as temporally (Fig. 3b). No attempt is made in this database to convert to a standardised mesh size and the mesh size is therefore included as a factor to be interpreted by the data users or used in statistical models.

In addition to the net-collected data, this database also contains 2256 zooplankton biomass records from CPR samples from on-going the Integrated Marine Observing System (IMOS) project (P_597). The CPR is towed primarily by “ships of opportunity” (commercial vessels plying their trading routes) as well as research vessels, at speeds of 10 to 25 knots and at ~10 m depth for distances up to 450 nautical miles per tow. Methods of counting and processing data are described by Richardson *et al*.^13^. The biomass estimate includes the dry weight of both the phytoplankton and zooplankton washed of the silk and filtered through a 75 μm filter, although the biomass is usually dominated by zooplankton. Further, the CPR underestimates zooplankton abundance and biomass compared with net-collected data sets^13,45,46^. Although conversions between CPR abundance (not biomass) and other sampling methods have been used before^47^, these are most likely species- and area-specific^13,48^. CPR data included in this dataset can therefore be used by themselves as they are internally consistent, but they are a poor measure of absolute abundance or biomass^13,48^.

Zooplankton biomass can also be estimated from measurements of plankton body-size by optical instruments. In this database, we include measurements from both the OPC^49^ and LOPC^15^ (P_1135). The optical instruments measure the change in light attenuance as particles pass in front of their light emitting diode (OPC) or laser (LOPC) light source. Changes in light attenuance are recorded as an Equivalent Spherical Diameter (ESD) which is the diameter of each particle, assuming each to be spherical in shape. As the many of the zooplankton (e.g. copepods) are the shape of an oblate spheroid (Length:Width:Depth ratio of 3:1:1)^49^, we use the cross-sectional area of the sphere to calculate the equivalent cross-sectional area of an oblate spheroid, giving an improved estimate of the length, width and depth measurements of the particle. These measurements, along with the density of water (1 g/mL), are used to calculate a biovolume and then biomass of each particle. The use of these instruments in oceanographic studies provides a standard procedure to rapidly count and size zooplankton at a fine spatial (metres) and temporal (0.5 seconds) resolution. However, there are limitations such as the lack of taxonomic information and the influence of sediment and marine snow^50^. The OPC and LOPC data in this dataset were collected from the *RV Southern Surveyor* and *RV Investigator* between the surface and a maximum depth of 300 m using a modified SeaSoar and Triaxus (MacArtney, Denmark). Each vertical cast was split into 50 m increments, and the summed biomass between ESDs of 300 μm and 12 mm reported^51,52^. As the OPC/LOPC data are derived from a measurement of individual size (rather than a direct biomass measurement) these data are not converted to a carbon equivalent and should be analysed separately unless standardised.

A total of 11573 records from net samples are included in the database. Of these data: 4434 records were from historical CSIRO datasets, 1835 records were provided by the Australian Institute of Marine Science (AIMS) from tropical Australian waters and 732 records were from on-going IMOS National Reference Stations project (P_599). On a global scale, Moriarty & O’Brien^33^ compiled 153,163 zooplankton biomass records in the online Coastal and Oceanic Plankton Ecology, Production and Observation Database (COPEPOD) hosted by NOAA (http://www.st.nmfs.noaa.gov/copepod). Only 2% of these COPEPOD records are within the geographical boundaries of 0–60°S, 110–160°E and are included within the Australian Zooplankton Biomass Database (P_1109), with acknowledgement (Online-only Table 1). Our data set contains >8000 additional net collected records of zooplankton biomass for the Australian region.

Unfortunately, while some of the earliest research cruises in the Australian region sampled zooplankton, they did not quantitatively measure the biomass^53^. This includes the following voyages/research expeditions: *Challenger* Expedition from 1873 to 1876 - off south-east Australia^54,55^; *Siboga* from 1899 to 1900 - around the Dutch East Indies^56^; British Antarctic Expedition *Terra Nova* from 1910 to 1913^57^; the Australia Antarctic Expedition from 1911 to 1914^58^; the 1928 GBR expedition^59,60^; *Dana II* from 1929 to 1930 in the Tasman Sea^61^; the BANZARE survey from 1929 to 1931 in the Australian-Antarctic quadrant^62^; Dakin and Colefax’s 1930s studies of New South Wales^63,64^; *NAGA* cruise S11 (cruises S1-S10 collected biomass but cruise S11 in Australian waters did not)^65^. The historical dataset with biomass data from *Discovery* from 1932 to 1934^66^ is included from COPEPOD along with datasets from 1956–2002. Details on individual projects included from COPEPOD are available from the links provided in Online-only Table 1.

## Data Records

In this database^6^, each data record belongs to a project (e.g. P_599 is the IMOS National Reference Stations dataset) and represents the total zooplankton biomass at a certain point in space and time and has been given a unique record identification number: P_(project_id) _(record_id). A project is defined as a set of data records that have been analysed together, usually as a cruise or study with the same sampling and processing methods. Metadata ascribed to a project relates to all the data records within that project (Online-only Table 1). Each sample within that project has a unique sample_id. The sample_id for each record is composed of a string of fields from the original data set to maintain traceability to the original source. Database fields for each data record are: latitude, longitude, sample date and time (local and UMT), depth (minimum, maximum, bottom_depth_measured and bottom_depth_ETOPO_estimate), biomass (value, measurement units and method) as in the original project, biomass converted to carbon (µgC/m^3^), net/gear type, mesh size, tow direction (vertical, horizontal, oblique) and day/night.

Most of the projects have ceased, and the final project dataset is collated for this database. However, the IMOS National Reference Stations (P_599) and Continuous Plankton Recorder Survey (P_597) datasets are ongoing long-term IMOS projects. Data are continually updated and available through the AODN separately. These two surveys from 2009 to the present, currently contribute 2988 records to the database and this figure will continue to increase.

## Technical Validation

Zooplankton biomass has been sampled using a variety of methods and measured in many ways. The biomass measurement method is recorded as the original measurement used by each project: biomass calculated as dry/wet mass or carbon; or biovolume calculated as displacement volume or settled volume. While projects are internally consistent, if the original values are to be used for analyses across projects, attention to the units of measurement is required as these vary (e.g. mg/m^3^ and g/m^3^). All biomass values (except from the optical dataset, P_1135) have been converted to a carbon equivalent (µg C/L) to allow inter-project comparison, but it is possible that the conversions (developed elsewhere in the world) do not hold true in some circumstances (e.g. high volumes of gelatinous specimens). If using multiple datasets, users are encouraged to build their own statistical models using the original projects as fixed or random factors.

Original data records with missing location and/or sampling dates have been excluded from the dataset. Some projects are missing data in some fields such as minimum and maximum depths, tow direction and/or net type. Using 1/6^th^ degree squares, the bottom_depth_ETOPO_estimate values from the ETOPO1 data is quite coarse and may be inaccurate for sample locations with rapidly changing bottom topography. For some records close to land, the ETOPO1 bathymetry could not be resolved for this dataset or was inaccurately calculated (e.g. Darwin Harbour samples: P_4). This is 125 records for the net data and 700 for the optical data; depth could be estimated from nautical charts if required. At 1/6^th^ degree squares, the ETOPO1 data is quite coarse and may be inaccurate for areas with rapidly changing bottom topography.

Day or night was calculated for each data record that had a time of collection, the records with no time have a blank. There are also some projects which had no time of collection provided but did note whether the sample was collected in day or night, so they also have the day_night data.

The optical plankton dataset (P_1135) provides biomass estimates from ESDs of 300 μm upwards. It has a collection aperture, but minimum organism size “sampled” is set by what is resolved by the instrument. The optical plankton biomass is an estimated wet weight calculated from the biovolume and these values are not directly comparable with the conventional net-collected biomass values. Optical samplers do not discriminate between particle types (i.e. between detritus and plankton), therefore the carbon equivalent is not calculated for this project dataset.

The CPR sampler (P_597) uses silk mesh, which retains smaller organism than conventional smoother nylon mesh^13^. We therefore suggest that the optical plankton and CPR data should be either analysed separately from the net-collected data or analysed together with net samples using appropriate statistical models. Note that the CPR and OPC/LOPC datasets have a high level of internal consistency.

As with the Australian Phytoplankton Database^8^, Zooplankton Abundance Database^7^, Chlorophyll *a* Database^9^, the Australian Zooplankton Biomass Database has been built with ease of use and minimising user error in mind. Therefore, it provides clean, easily comparable data at a level that requires minimal interpretation. The CSIRO database holds all the raw data, original datasets and ambiguous records from the constituent datasets, and researchers can request further information from the custodians detailed in Online-only Table 1. In this database, the COPEPOD datasets include the “copePID”: a permanent unique identifier to link to each project data set in COPEPOD, this is the first 8 digits of the sample_id. This enables access to the dataset and associated metadata in COPEPOD (e.g. au-04001: https://www.st.nmfs.noaa.gov/copepod/data/au-04001/).

## Usage Notes

With a comprehensive continental-scale dataset, care must be taken with analysis as the data were collected using very different methods. Data were collected under vastly different environmental conditions, from tropical, to sub-Antarctic waters.

This database^6^ can be used in conjunction with the Australian Zooplankton Abundance Database^7^, which provides species-level data and is available through the AODN. The Australian Phytoplankton Abundance Database^8^ and the Australian Chlorophyll a Database^9^ (both also available through the AODN) provide complementary data that can be matched to the zooplankton biomass data via Project_id and to individual records via sample_id or by sampling date and time details. The Australian Larval Fish database^67^ also includes information from Project P_599: the IMOS National Reference Stations and is also available for comparison through the AODN.

## Online-only Table

**Online-only Table 1:** Overview of the datasets in the database by project showing location, dates, biomass measurement method, sampling gear details, and number of records.

Project id	Project name	Sampling location	Custodian	Custodian afflilation	Sampled years	Depth range (m)	Biomass measurement method	Original biomass units	Sampler method	Net mesh size (µm)	Tow direction	Number of records	Acknowledgement	Reference number
P_2	Lihir effects of mining	Papua New Guinea	Anthony Richardson	CSIRO	2006–2006	0–500	dry mass	mg/m^3^	MIDOC	150	oblique	196		^68^
P_3	Ramu effects of mining	Papua New Guinea	Anthony Richardson	CSIRO	2009–2009	50–250	dry mass	mg/m^3^	Ring net	150	oblique	34		^69^
P_4	Darwin Harbour zooplankton	Darwin, Northern Territory, Australia	David Mckinnon	AIMS	2002–2004	0–30	dry mass	mg/m^3^	Ring net	73	vertical	56		^70^
P_5	Kimberley zooplankton (150 micron)	Kimberley, Western Australia, Australia	David Mckinnon	AIMS	2011–2014	0–77	dry mass	mg/m^3^	Bongo net	350	oblique	88		^71^
					2011–2014	0–77	dry mass	mg/m^3^	Bongo net	73	oblique	88		
					2011–2014	0–77	dry mass	mg/m^3^	Bongo net	150	oblique	87		
					2014–2014	0–18	dry mass	mg/m^3^	WP-2 (UNESCO Working Party 2)	150	oblique	1		
P_7	Scott Reef ecosystem processes, zooplankton (100 micron)	Scott Reef, Western Australia, Australia	David Mckinnon	AIMS	2008–2009	to 400	dry mass	mg/m^3^	Multi net	100	vertical	138		^72^
P_8	Scott Reef ecosystem processes, zooplankton (500 micron)	Scott Reef, Western Australia, Australia	David Mckinnon	AIMS	2009–2009	to 175	dry mass	mg/m^3^	Multi net	500	vertical	3		^72^
P_9	Bathurst Island	Bathurst Island, Northern Territory, Australia	David Mckinnon	AIMS	2005–2006	0–10	dry mass	mg/m^3^	Ring net	73	vertical	6		^73^
P_10	Albatross Bay zooplankton	Gulf of Carpentaria, Australia	Peter Rothlisberg	CSIRO	1986–1988	0–60	dry mass	mg/m^3^	Plankton Net 0.5*0.5m square frame & multi-vane steel kite-otter depressor	142	oblique	374		^21^
					1988–1992	0–69	dry mass	mg/m^3^	Plankton Net 0.5*0.5m square frame & multi-vane steel kite-otter depressor	250	oblique	1166		
P_11	WA Eddies	Mid-west coast of Australia	Joanna Strzelecki & Tony Koslow	CSIRO	2003–2003	0–150	dry mass	mg/m^3^	Bongo net	355	double	8		^74^
P_15	Zooplankton dynamics in the GBR	Great Barrier Reef, Queensland, Australia	David Mckinnon	AIMS	2000–2002	0–40	dry mass	mg/m^3^	Ring net	73	vertical	42		^75^
P_16	Wessels, Arafura Sea	Arafura Sea, Australia	David Mckinnon	AIMS	2011–2011	0–53	dry mass	mg/m^3^	Bongo net	100	oblique	11		^76^
P_17	North West Cape	North West Cape, Western Australia, Australia	David Mckinnon	AIMS	1997–2003	0–75	dry mass	mg/m^3^	Ring net	73	vertical	172		^77^
P_21	East coast Tasmania, shelf and near-shelf waters	Mid- east coast Tasmania, Australia	Kerrie Swadling	IMAS	1971–1972	0–20	dry mass	mg/100m^3^	WP-2 (UNESCO Working Party 2)	200	horizontal	92		^78^
P_23	Mid-slope zooplankton south of Tasmania	Southern Ocean, south of Tasmania, Australia	Kerrie Swadling	IMAS	1992–1993	0–900	dry mass	mg/m^3^	EZ	335	oblique	4		^79^
P_591	Moreton Bay plankton community dynamics	Moreton Bay, Queensland, Australia	Sarah Pausina	UQ	2009–2012	0.2–0.2	dry mass	mg/m^3^	Ring net	100	horizontal	89	Healthy Waterways ARC LPO883663	^80^
P_597#	IMOS AusCPR	Australia	Anthony Richardson	CSIRO	2010–2019	5–10	dry mass	mg/m^3^	Continuous plankton recorder	270	oblique	2256	IMOS	^13^
P_599	IMOS National Reference Stations	Darwin, Esperance, Kangaroo Island, Maria Island, Ningaloo, North Stradbroke Island, Yongala, Australia	Anthony Richardson	CSIRO	2008–2019	0–110	dry mass	mg/m^3^	Drop net	100	vertical	732	IMOS	^39,81^
P_1088	Zooplankton of Lady Elliot Island	Lady Elliot Island, Great Barrier Reef, Queensland, Australia	Asia Armstrong	UQ	2014–2014	0–2	wet mass	mg/m^3^	Ring net	200	horizontal	90	UQ	^82^
P_1089	Coral Sea zooplankton	Coral Sea, Australia	David Mckinnon	AIMS	2011–2013	0–150	dry mass	mg/m^3^	Ring net	73	vertical	47	AIMS	^26,83^
					2012–2014	0–200	dry mass	mg/m^3^	Bongo net	73	oblique	33	AIMS	
					2012–2014	0–200	dry mass	mg/m^3^	Bongo net	350	oblique	32	AIMS	
					2012–2014	0–200	dry mass	mg/m^3^	Bongo net	150	oblique	33	AIMS	
P_1090	CSIRO OCR cruises 1959-62	Australia	CSIRO_literature	CSIRO	1959–1962	0–400	wet mass	mg/m^3^	Modified Clarke-Bumpus Sampler	270	oblique	272	CSIRO O&A Data Centre	^84–93^
					1960–1961	0–400	wet mass	mg/m^3^	Modified Clarke-Bumpus Sampler	270	horizontal	70		
					1961–1961	0–200	wet mass	mg/m^3^	JUDAY NET (type not specified)	170	vertical	3		
					1961–1961	0–0	wet mass	mg/m^3^	SCRIPPS High Speed Sampler	330	horizontal	44		
P_1091	Derwent Hunter OSL cruises 1960-62	East coast of Australia	CSIRO_literature	CSIRO	1960–1962	0–250	wet mass	mg/m^3^	Modified Clarke-Bumpus Sampler	260	oblique	150	CSIRO O&A Data Centre	^94–97^
					1961–1961	0–50	wet mass	mg/m^3^	Modified Clarke-Bumpus Sampler	260	horizontal	21		
P_1092	Derwent Hunter cruises 1957	East coast of Australia	CSIRO_literature	CSIRO	1957–1957	0–200	displacement volume	ml/m^3^	Clarke-Bumpus Sampler	270	oblique	44	CSIRO O&A Data Centre	^98–100^
P_1098	Tropical Australia zooplankton (63 micron) 1987-2009	Australia	Miles Furnas	AIMS	1987–2005	0–75	dry mass	mg/m^3^	Ring net	73	vertical	821		^83^
P_1099	Zooplankton biomass in GOC 1975-77	Gulf of Carpentaria, Australia	CSIRO O&A Data Centre	CSIRO	1975–1977	0–66	dry mass	mg/m^3^	Plankton Net 0.5*0.5m square frame & multi-vane steel kite-otter depressor	142	oblique	300	CSIRO O&A Data Centre	^101^
P_1100	Southern Surveyor cruises Griffiths	Australia	CSIRO O&A Data Centre	CSIRO	1991–1993	0–100	settled volume	ml/m^3^	Drop net	200	vertical	104	CSIRO O&A Data Centre	^102^
P_1101	Inshore GBR waters 1976-78	Great Barrier Reef, Queensland, Australia	C.Lonborg	AIMS	1976–1978	0–40	wet mass	mg/m^3^	Conical net	205	vertical	177	AIMS	^103^
P_1105	Tranter zooplankton cruises 1957-63	Australia	CSIRO	CSIRO	1957–1961	to 200	wet mass	mg/m^3^	Modified Clarke-Bumpus Sampler	270	oblique	756		^104^
					1959–1961	0–126	wet mass	mg/m^3^	Modified Clarke-Bumpus Sampler	270	horizontal	287		
P_1106	BIONESS cruises 1992-93	East coast of Australia	Jock Young	CSIRO	1992–1993	0–400	dry mass	mg/m^3^	EZ	335	oblique	82		^105^
P_1108	Australian Equatorial JGOFS dataset	North coast of Australia	CSIRO	CSIRO	1992–1993	0–100	settled volume	ml/m^3^	Drop net	200	vertical	42		^106^
P_1109	COPEPOD:au-04001 Magga Dan, 1959	Australia	Todd O'Brien	NOAA	1959–1959	0–100	wet mass	mg/m^3^	Clarke-Bumpus Sampler	300	vertical	5	COPEPOD NOAA; CSIRO Australia	
P_1109	COPEPOD:au-05101 CSIRO Australia, 1959-1963	Australia	Todd O'Brien	NOAA	1959–1963	0–400	wet mass	mg/m^3^	Clarke-Bumpus Sampler	318	oblique	731	COPEPOD NOAA; CSIRO Australia	
					1960–1960		wet mass	mg/m^3^	Clarke-Bumpus Sampler	(null)	unknown	1	http://www.st.nmfs.noaa.gov/copepod	
					1961–1961	0–100	dry mass	mg/m^3^	Clarke-Bumpus Sampler	318	oblique	12		
					1961–1961	0–250	wet mass	mg/m^3^	Clarke-Bumpus Sampler	300	oblique	38		
					1961–1961	0–100	wet mass	mg/m^3^	Clarke-Bumpus Sampler	318	horizontal	21		
					1961–1962		wet mass	mg/m^3^	Clarke-Bumpus Sampler	318	unknown	9		
					1962–1963	0–200	wet mass	mg/m^3^	Indian Ocean Standard Net (IOSN)	330	vertical	287		
P_1109	COPEPOD:fr-03004 L'ORSTOM III, 1958	Australia	Todd O'Brien	NOAA	1958–1958	0–381	displacement volume	ml/m^3^	Plankton Net (type not specified)	366	oblique	6	COPEPOD NOAA; Oceanic Lab of the Inst of French Oceania, Noumea - New Caladonia	
P_1109	COPEPOD:fr-03103 CINECA IV - CORINDON - CORiolis-INDONesia, 1981	Australia	Todd O'Brien	NOAA	1981–1981	0–0	settled volume	ml/m^3^	WP-2 (UNESCO Working Party 2)	200	vertical	29	COPEPOD NOAA; Office de la Recherche Scientifique et Technique Outre Mer (ORSTOM), France	
					1981–1981	0–0	wet mass	mg/m^3^	WP-2 (UNESCO Working Party 2)	200	vertical	29	http://www.st.nmfs.noaa.gov/copepod	
P_1109	COPEPOD:id-05101 IMR Collection,1970-1983	Australia	Todd O'Brien	NOAA	1970–1976	to 350	settled volume	ml/m^3^	NORPAC (North Pacific Standard Net)	333	vertical	123	COPEPOD NOAA; Institute of Marine Research-Jakata (IMR), Indonesia	
					1970–1983	to 350	displacement volume	ml/m^3^	NORPAC (North Pacific Standard Net)	333	vertical	629	http://www.st.nmfs.noaa.gov/copepod	
					1971–1971	0–100	settled volume	ml/m^3^	unknown	118	vertical	49		
					1971–1973	0–161	settled volume	ml/m^3^	Plankton Net (type not specified)	119	vertical	74		
					1972–1983	0–199	settled volume	ml/m^3^	unknown	119	vertical	297		
					1973–1973	0–0	displacement volume	ml/m^3^	NORPAC (North Pacific Standard Net)	333	oblique	1		
					1973–1973	0–100	settled volume	ml/m^3^	NORPAC (North Pacific Standard Net)	119	vertical	25		
					1974–1974	0 to unknown	displacement volume	ml/m^3^	unknown	515	horizontal	18		
					1974–1974	0 to unknown	settled volume	ml/m^3^	unknown	515	horizontal	17		
					1976–1976	0–100	settled volume	ml/m^3^	Kitahara(n) Net	118	unknown	1		
					1976–1976	0–100	settled volume	ml/m^3^	Kitahara(n) Net	333	unknown	19		
					1977–1977	0–126	settled volume	ml/m^3^	Kitahara(n) Net	120	vertical	21		
					1979–1979	0–200	displacement volume	ml/m^3^	NORPAC (North Pacific Standard Net)	333	unknown	16		
					1979–1979	0–1058	settled volume	ml/m^3^	unknown	119	unknown	23		
					1980–1982	0–100	settled volume	ml/m^3^	unknown	75	vertical	27		
P_1109	COPEPOD:jp-04301 Hakuho Maru collection, 1979	Australia	Todd O'Brien	NOAA	1979–1979	0–151	settled volume	ml/m^3^	NORPAC (North Pacific Standard Net)	100	vertical	7	COPEPOD NOAA; Japan Meteorological Agency (JMA), Japan	
					1979–1979	0–151	settled volume	ml/m^3^	NORPAC (North Pacific Standard Net)	350	vertical	7	http://www.st.nmfs.noaa.gov/copepod	
					1979–1979	0–151	wet mass	mg/m^3^	NORPAC (North Pacific Standard Net)	100	vertical	7		
					1979–1979	0–151	wet mass	mg/m^3^	NORPAC (North Pacific Standard Net)	350	vertical	7		
P_1109	COPEPOD:jp-05101 Japanese Antarctic Research Expedition (JARE), 1972–2008	Australia	Todd O'Brien	NOAA	1972–1996	0–200	wet mass	mg/m^3^	NORPAC (North Pacific Standard Net)	330	vertical	31	COPEPOD NOAA; National Institute of Polar Research – (NIPR), Japan	
					1987–1996	0–200	wet mass	mg/m^3^	NORPAC (North Pacific Standard Net)	110	vertical	22	http://www.st.nmfs.noaa.gov/copepod	
					1996–2008	0–150	wet mass	mg/m^3^	Twin NORPAC Net	110	vertical	38		
					1996–2008	0–150	wet mass	mg/m^3^	Twin NORPAC Net	330	vertical	37		
P_1109	COPEPOD:jp-05201 JMA North Pacific Surveys, 1993-2002	Australia	Todd O'Brien	NOAA	1999–2002	0–160	wet mass	mg/m^3^	NORPAC (North Pacific Standard Net)	330	oblique	14	COPEPOD NOAA; Japan Meteorological Agency (JMA), Japan	
P_1109	COPEPOD:nl-03001 Snellius II Expedition, 1984-1985	Australia	Todd O'Brien	NOAA	1984–1985	0–240	displacement volume	ml/m^3^	Gulf V Sampler (modified Gulf III; high speed)	200	oblique	52	COPEPOD NOAA; Netherlands Council of Oceanic Research (NRZ), Netherlands	
					1984–1985	0–192	displacement volume	ml/m^3^	NORPAC (North Pacific Standard Net)	150	vertical	43	http://www.st.nmfs.noaa.gov/copepod	
					1984–1985	0–193	displacement volume	ml/m^3^	NORPAC (North Pacific Standard Net)	300	vertical	44		
					1984–1985	0–193	displacement volume	ml/m^3^	NORPAC (North Pacific Standard Net)	500	vertical	42		
					1984–1985	0–160	displacement volume	ml/m^3^	Plankton Net (type not specified)	200	vertical	39		
					1984–1985	0–122	settled volume	ml/m^3^	Kitahara(n) Net	75	vertical	44		
P_1109	COPEPOD:ru-01003 Soviet Antarctic Expedition (SAE) - R/V OB 1956–1958	Australia	Todd O'Brien	NOAA	1956–1958	0–600	settled volume	ml/m^3^	JUDAY Net 37/50	168	vertical	11	COPEPOD NOAA; Arctic and Antarctic Scientific Research Institute (AARI), Zoological Institute Russian Academy of Science, Russia	
					1958–1958	0–500	settled volume	ml/m^3^	K100 Net	300	vertical	1	http://www.st.nmfs.noaa.gov/copepod	
P_1109	COPEPOD:ru-04102 Vityaz Indian Ocean Cruises 31 and 33, 1959	Australia	Todd O'Brien	NOAA	1959–1959	0–500	wet mass	mg/m^3^	JUDAY 80/113 Oceanic Model	168	vertical	112	COPEPOD NOAA; Institute of Oceanology/USSR Academy of Sciences – Vityaz Indian Ocean Cruises	
P_1109	COPEPOD:ru-04103 Vityaz Pacific Ocean Cruises, 1957-1958	Australia	Todd O'Brien	NOAA	1957–1957	0–100	wet mass	mg/m^3^	JUDAY Net 37/50	168	vertical	13	COPEPOD NOAA; Institute of Oceanology/USSR Academy of Sciences – Vityaz Pacific Ocean Cruises	
					1958–1958	0–532	displacement volume	ml/m^3^	JUDAY Net 37/50	168	vertical	19	http://www.st.nmfs.noaa.gov/copepod	
P_1109	COPEPOD:uk-04102 Foxton 1956, Discovery Cruises - Volume XXVIII, 1932-1939	Australia	Todd O'Brien	NOAA	1932–1939	0–1500	displacement volume	ml/m^3^	Bongo net + Multi net	240	vertical	137	COPEPOD NOAA; National Institute of Oceanography, United Kingdom	
P_1109	COPEPOD:us-01048 SOSC Gear Study, 1967	Australia	Todd O'Brien	NOAA	1967–1967	0–50	displacement volume	ml/m^3^	ICITA plankton net	281	vertical	31	COPEPOD NOAA; Smithsonian Institution, USA	
					1967–1967	0–50	displacement volume	ml/m^3^	Indian Ocean Standard Net (IOSN)	333	vertical	14		
P_1109	COPEPOD:us-04101 R/V ELTANIN Cruises 16, 25, 26, 27, 1965-1969	Australia	Todd O'Brien	NOAA	1965–1965	0–250	displacement volume	ml/m^3^	Be' Multiple Plankton Sampler (MPS)	200	vertical	1	COPEPOD NOAA; United States Antarctic Research Project - USAP/USARP, USA	
					1965–1965	0–10	displacement volume	ml/m^3^	Plankton Net (type not specified)	200	horizontal	1	http://www.st.nmfs.noaa.gov/copepod	
					1965–1965	0–250	displacement volume	ml/m^3^	Plankton Net (type not specified)	200	oblique	1		
					1965–1965	0–1000	displacement volume	ml/m^3^	Plankton Net (type not specified)	200	vertical	2		
					1967–1967	500–2000	settled volume	ml/m^3^	Bathypelagic Plankton Sampler (BPS)	200	vertical	2		
					1967–1967	0–500	settled volume	ml/m^3^	Be' Multiple Plankton Sampler (MPS)	200	vertical	5		
					1967–1967	0–10	settled volume	ml/m^3^	Plankton Net (type not specified)	200	horizontal	11		
					1967–1967	0–2000	settled volume	ml/m^3^	Plankton Net (type not specified)	200	vertical	20		
P_1109	COPEPOD:zz-03201 International Indian Ocean Expedition (IIOE), 1962-1965	Australia	Todd O'Brien	NOAA	1962–1962	0–260	displacement volume	ml/m^3^	JUDAY 80/113 Oceanic Model	170	vertical	1	COPEPOD NOAA	
					1962–1965	0–560	displacement volume	ml/m^3^	Indian Ocean Standard Net (IOSN)	330	vertical	212	http://www.st.nmfs.noaa.gov/copepod	
					1968–1968	0–125	wet mass	mg/m^3^	NORPAC (North Pacific Standard Net)	330	vertical	1		
P_1110	GOC & GAB productivity 1965 & 1968	Gulf of Carpentaria & Great Australian Bight, Australia	Motoda	Hokkaido University	1965–1968	0–144	wet mass	mg/m^3^	NORPAC (North Pacific Standard Net)	350	vertical	38		^107^
P_1112	Plankton at Cronulla onshore station	East coast of Australia	CSIRO	CSIRO	1943–1946	0–0	displacement volume	ml/m^3^	N70 Discovery Net Horizontal	240	horizontal	133		^108^
P_1115	Marmion Lagoon zooplankton	Mid-west coast of Australia	Joanna Strezlecki	CSIRO	2007–2008		dry mass	g/m^3^	Bongo net	100	oblique	38		^109^
P_1135*	LOPC around Australia	Australia	Jason Everett & Iain Suthers	UNSW	2004–2018	2–600	Estimated Wet Weight from BioVolume	mg/m^3^	Optical Plankton Counter (OPC-2T)	300	oblique	35358		^52,53^
P_1142	East Indian Ocean larval research 1987	East Indian Ocean	CSIRO	CSIRO	1987–1987	0–2	settled volume	ml/m^3^	Ring net	500	horizontal	179		^110^
					1987–1987	0–40	settled volume	ml/m^3^	Ring net	500	oblique	5		
					1987–1987	0–50	settled volume	ml/m^3^	Ring net	500	double	104		
					1987–1987	0–79	settled volume	ml/m^3^	Bongo net	500	oblique	14		
P_1143	Eastern GAB productivity	Great Australian Bight, Australia	Paul van Ruth	SARDI	2004–2005	0–370	settled volume	ml/m^3^	Conical net	150	vertical	39	SARDI	^111^
P_1144	Soela cruises GAB 1980-81	Great Australian Bight, Australia	CSIRO	CSIRO	1980–1981	0–250	settled volume	ml/m^3^	Drop net	200	vertical	152		^112^
P_1148	Storm Bay study 1985-87	Tasmania, Australia	Lesley Clementson	CSIRO	1985–1988	0–40	settled volume	ml/m^3^ log normal	Drop net	200	vertical	132		^113^
P_1149	Zooplankton biomass in Storm Bay	Tasmania, Australia	Kerrie Swadling & Christine Crawford	IMAS	2009–2015	0–90	settled volume	ml/m^3^	Bongo net	200	vertical	407		^114^

Note: P_597 is Continuous Plankton Recorder data, P_1135 is optical data . The other projects all used tradional nets for sample collection.
